# Supplementary material for: Antimicrobial and Synergistic Effects of Commercial Piperine and Piperlongumine in Combination with Conventional Antimicrobials
Source: Antibiotics (Basel). 2019 May 4;8(2):55. doi: 10.3390/antibiotics8020055 (PMC6627571; doi:10.3390/antibiotics8020055)
Supplement: Supplementary File 1 [file antibiotics-08-00055-s001.pdf]

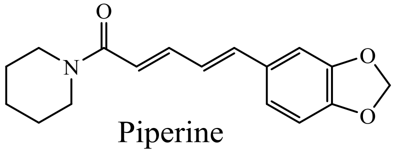

Piperine

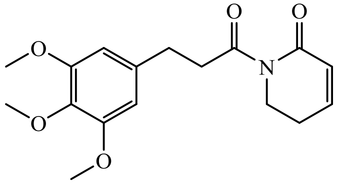

Piperlongumine

+

Conventional antimicrobials (rifampicin, tetracycline and itraconazole)

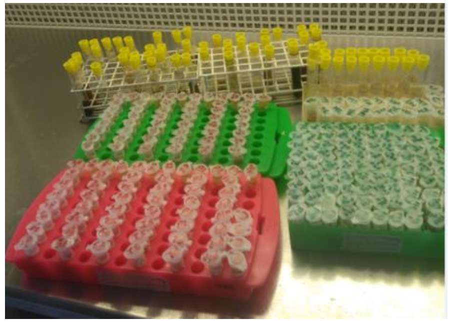

Synergistic evaluation against
S. aureus, P. aeruginosa and C. albicans

Synergistic effect is dependent on the ratio of combination of piperamide/antimicrobial.
